# Current Therapies for Cholestatic Diseases

**DOI:** 10.3390/biomedicines11061713

**Published:** 2023-06-15

**Authors:** Nahum Méndez-Sánchez, Carlos E. Coronel-Castillo, Ana L. Ordoñez-Vázquez

**Affiliations:** 1 Unit Liver Research, Medica Sur Clinic & Foundation, Puente de Piedra 150, Toriello Guerra, Tlalpan, Mexico City 14050, Mexico; annordonez.07@hotmail.com; 2 Faculty of Medicine, National Autonomous University of Mexico, Av. Universidad 3004, Copilco Universidad, Coyoacán, Mexico City 04510, Mexico; 3 Internal Medicine Section, Central Military Hospital, Manuel Ávila Camacho s/n, Militar, Miguel Hidalgo, Ciudad de México 11200, Mexico; carlos-cc@outlook.com

**Keywords:** cholestasis, bile acids, ursodeoxycholic acid, fibrosis, FXR agonist, PXR agonist, fibreates

## Abstract

Cholestasis is a condition characterized by decrease in bile flow due to progressive pathological states that lead to chronic cholestatic liver diseases which affect the biliary tree at the intrahepatic level and extrahepatic level. They induce complications such as cirrhosis, liver failure, malignancies, bone disease and nutritional deficiencies that merit close follow-up and specific interventions. Furthermore, as those conditions progress to liver cirrhosis, there will be an increase in mortality but also an important impact in quality of life and economic burden due to comorbidities related with liver failure. Therefore, it is important that clinicians understand the treatment options for cholestatic liver diseases. With a general view of therapeutic options and their molecular targets, this review addresses the pathophysiology of cholangiopathies. The objective is to provide clinicians with an overview of the safety and efficacy of the treatment of cholangiopathies based on the current evidence.

## 1. Introduction

Cholestasis is a condition characterized by a decrease in bile flow due to progressive pathological states that lead to chronic cholestatic liver diseases (CLD) which affect the biliary tree at the intrahepatic level and extrahepatic level [1]. Those conditions share commons mechanism and etiologies, either genetical or immunological. The two most prevalent of CLD are primary biliary cholangitis (PBC) and primary sclerosing cholangitis (PSC) [2,3].

Cholestasis induces symptoms such as pruritus and fatigue but also important complications such as cirrhosis, liver failure, malignancies, bone disease and nutritional deficiencies that merit close follow-up and specific interventions to improve quality of life [4]. As CLD progress to liver cirrhosis, there will be an increase in mortality but also an important impact in quality of life and economic burden due to comorbidities related with liver failure. CLD represent high costs to patients because of a reduction in outpatient activities but also from hospitalizations, mostly due to cirrhosis complications and liver transplantation (LT) [5]. In a study carried out from 1988 to 2018, CLD, and particularly PBC and PSC, accounted for 14.2% of LT. About 10% to 40% of those patients will have a recurrence of primary disease after LT [6]. The aim of this review is to offer clinicians a review of the updated literature on the treatment of cholestasis and the associated complications.

## 2. Pathophysiology of Cholangiopathies

**Figure 1:**
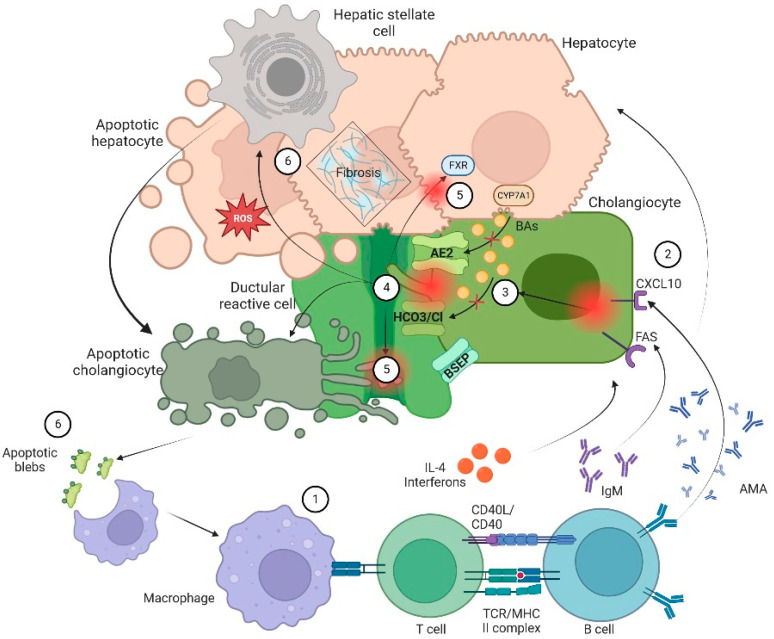
Pathophysiology of cholangiopathies. In general, patients with CLD are in a proinflammatory state due to an exposition to a microbial and immunogenic environment with mitochondrial autoantigens in the form of apoptotic blebs that induce an immune response. (1) Dysregulation of the innate and adaptive immune response results in directed damage to cholangiocytes. (2) Small cholangiocytes act as antigen presenting cells and produce a variety of cytokines such as IL-6 and IL-8 and increase the expression of CXCL10. The latter will allow an increase in the production of IFNγ and other chemoattractants that result in T-cell infiltration and production of anti-mitochondrial antibody by B cells that ultimately increase production of bile. On the other hand, (3) there is a failure of biliary transporters (AE2, BSEP and the HCO3 umbrella) due to direct damage of cytotoxic T cells with mitochondrial injury on biliary cells. Hence, cholangiocytes lose their protection against toxic hydrophobic BAs and reduce BAs transportation in the context of bile overproduction. (4) Inflammation and injury lead to ductular reaction (DR), infiltration of leukocytes and lymphocytes that activate liver progenitor cells, thus increasing matrix protein levels through different proteins such as TGFβ1/2. Moreover, bile salts will be acidified becoming hydrophobic; eventually these toxic bile salts cross the membrane leading to apoptosis. (5) In addition, the constant bile duct insult led to retention of hydrophobic Bas promoting local injury and altering the enterohepatic circulation of bile. Finally, there is a dysregulation in FXR activation that may lead to a profibrotic state. (6) Apoptosis and cell injury promotes formation of reactive oxygen species (ROS) both in liver cells and cholangiocytes. Furthermore, apoptosis releases apoptotic blebs that perpetuate a proinflammatory state. All those processes induce cells to evolve toward an exhausted, profibrogenic phenotype which can contribute to the development of hepatic stellate cell-mediated liver fibrosis.

The general sequence of events leading to cholestasis and its associated damage can be summarized as: (1) ductular reaction (DR), (2) biliary stasis (intrinsic and/or extrinsic obstruction), (3) modification of bile components to a cytotoxic profile and (4) a proinflammatory and profibrotic state. It is essential to know biology of cholangiocytes and the general aspects of pathophysiology to understand the therapeutic targets of the different pharmacological options (Figure 1).

### 2.1. Cholangiocytes Biology

Cholangiocytes are the metabolically active epithelium that lines the bile ducts, accounting for 3% to 5% of the total of liver cells. Furthermore, cholangiocytes are heterogeneous both in functions and morphology. For instance, small cholangiocytes possess an especial plasticity under stress conditions. In fact, small cholangiocytes are more resistant to injury than large cholangiocytes and proliferate instead of dying, unlike the large ones. On the other hand, large cholangiocytes are involved in bile secretion and are characterized by the expression of anion exchangers, bile acids (BAs) transporters and secretin receptors. Cholangiocytes express water channels such as aquaporins, transporters such as the sodium/glucose cotransporter 1 (SGLT1) and important exchangers, mainly the Cl^−^/HCO^3−^ exchanger. Therefore, the main functions of cholangiocytes consist of bile production, regulation of bile volume and composition and their transport. They are also involved in modulation of liver injury and the repairing mechanism [6,7].

Regarding bile production, about 40% of the daily bile output is made by cholangiocytes. That process starts when secretin binds to its receptor on the basolateral membrane of cholangiocytes and activates the cyclic adenosine monophosphate (cAMP) signaling pathway which in turn activates the apical chloride channels leading to a release of chloride ions that drives bicarbonate secretion by activating the AE2 chloride/bicarbonate exchanger helping to the alkalinity of bile. In the case of bile output and BAs transport, it is important to notice that cholangiocytes are exposed to high concentrations of bile on their apical side. BAs are transported into cholangiocytes through the apical sodium-dependent bile acid transporter (ASBT) on their apical membrane; once inside, BAs can stimulate a secretin-induced cAMP secretory response as well as stimulate bicarbonate secretion, hence regulating bile flow [7].

In addition to the latter functions, cholangiocytes have important immune activity, as they express and secrete interleukins 6 (IL-6) and 8 (IL-8), and monocyte chemotactic protein-1 (MCP1) through the TLR4-NF-κB and TLR4-MAPK signaling pathway which is initiated by proinflammatory cytokines such as TNF-α or by bacterial products. Moreover, cholangiocytes interact with CD4+ and CD8+ T cells via IL-8 and MCP1, but also by expressing adhesion molecules. It is possible that cholangiocytes may act as antigen presenting cells since they also express major histocompatibility complex class I and class II. Thus, cholangiocytes not only regulate bile metabolism and transport, but act as inflammatory mediators in CLD and play an important role in the progression of liver fibrosis [6,7,8].

### 2.2. Ductular Reaction

As stated before, cholangiocytes participate in the formation and modification of bile through a series of transmembrane channels, transporters and exchangers that are expressed in the apical or basolateral domain [7]. There are multiple triggers or promoters in the activation of cholangiocytes or DR in which the number of ductules increases, accompanied by infiltration of leukocytes and lymphocytes, activation of liver progenitor cells and an increase in matrix protein levels [8]. Initially, DR is a response to an aggressor stimulus; it tries to limit and eliminate the harmful factor. If DR is perpetuated, proinflammatory pathways (Notch and Hedgehog) induce cholangiocytes maturation, fibronectin deposition and cytokine transcription (interleukin-6, interleukin-8, tumor necrosis factor and various growth factors) that generate histological and structural changes that condition cholestasis. Furthermore, cholangiocytes recognize the presence of pathogens through pattern recognition receptors (PRR), and the activation of these PRRs triggers a signaling cascade which results in the expression of various cytokines, immunoglobulins, adhesion molecules and a strong cellular response (CD4+ T cells, CD8+ T cells, B cells, macrophages, and natural killer cells) that can release pro- and anti-inflammatory molecules and an angiogenic, fibrogenic and proliferative factor. This is the basis for consideration of immunomodulatory therapies for CLD [9].

### 2.3. Biliary Stasis

Biliary stasis is due to impaired bile flow and impaired secretion by hepatocytes. An altered transport is another predisposing factor for cholestatic status, RD, increase BAs concentration and mitochondrial dysfunction [6]. The dysfunction of apical and basolateral cotransporters of the cholangiocytes generates cholestasis impairing secretion by the canalicular membrane.

Primary BAs are synthesized in the liver where they undergo conjugation to form bile salts and are then secreted in the bile canaliculi to reach the intestine where primary bile salts undergo dehydroxylation and deconjugation by the intestinal microbiota and form secondary BAs. BAs are then passively reabsorbed in the upper intestine and terminal ileum via ASBT. Once reabsorbed, BAs are transport via the organic solute transporter (OST) α/β to the portal circulation, thus returning to the hepatocyte by other two transporters, either the sodium-taurocholate cotransporter polypeptide (NTCP) or the organic anion transporter polypeptide (OATP). In addition, the enterohepatic circulation of BAs is regulated by different feedback mechanisms that protect hepatocytes from BAs cytotoxicity, which is the most important the negative feedback of the nuclear farnesoid X receptor (FXR).

**Figure 2:**
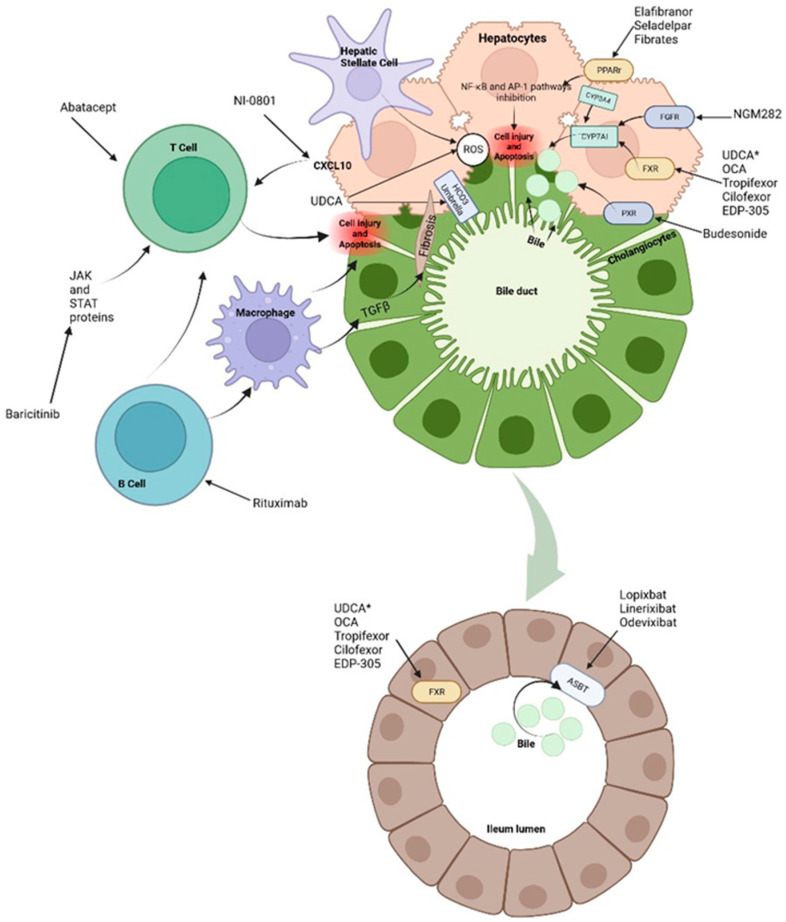
Treatments and molecular targets. In the liver, FXR agonists, PPAr agonist and FGF agonist improve cholestasis by decreasing BAs production and output via CYP7A1 inhibition. PPAr agonist and FRX also modulate the proinflammatory state by suppression of inflammatory pathways such as NF-kB. This results in ROS and interleukin reduction as well as decrease in hepatic stellate cells and immune cells activation. Immunomodulatory therapies, such as Rituximab or Baricitinib are aimed at blocking antigen presentation and the direct damage to liver cells. It is important to note that apoptosis releases apoptotic blebs which perpetuates a proinflammatory state and the formation ROS both in liver cells and cholangiocytes. Among other mechanisms, * UDCA serves as a partial agonist of FXR, improves BAs flow and enhances ions channels. Its effects on the biliary HCO^3−^ umbrella, near the apical surface, prevents the permeation of hydrophobic BAs; hence, contributing to the reduction in cell injury. Intestinal FXR agonists enhance the alleviation of cholestasis in the digestive system by reducing the overall size of the BAs pool that is reabsorbed. The events triggered by FXR in the gut liver axis probably represent a therapeutic option through the decrease in the excess of BAs in the liver. Finally, about 90–95% of BAs are reabsorbed into the terminal ileum via ASBT. Hence, the inhibition of ASBT reduces the overload of BAs in the liver. The decrease in ASBT expression increases the excretion of fecal BAs and the total concentration of BAs in liver.

In the enterocytes, BAs bind to FXR to decrease its synthesis via CYP7A1. This pathway can also be suppressed by the activity of fibroblast enteral growth factor 19 (FGF-19) that binds to its hepatocyte receptor and by fibroblast growth factor 4/β-klotho (FGFR4/β-klotho) complex to inhibit transcription of the CYP7A1 gene. FGF19 is an endocrine gastrointestinal hormone that controls the metabolism of BAs through its effects on CYP7A1, which is the first and rate-limiting enzyme in the classic pathway of BAs’ synthesis [10]. In cholestasis, high levels of BAs induce FGF-19 expression. The increased concentration of FGF-19 in the gut stimulates activation of the FGFR4/β-klotho receptor in the liver which is associated with malignancy risk [11]. On the other hand, FXR activation leads to downregulation of the intestinal BAs transporter ASBT, the hepatic uptake transporters NTCP and OATP, and upregulation of bile salt export pump (BSEP) of hepatic efflux transporters (Figure 1) (Figure 2) [6,7,8,9].

### 2.4. Citotoxic Profile of Biliary Acids

The leakage and accumulation of BAs in hepatocytes at notably high concentrations can trigger the activation of cholangiocytes, resulting in chronic inflammation, proliferation, apoptosis, and, as the damage progresses, fibrosis [1,2,3,4,5,6,7,8,9]. BAs are composed of four steroid rings that form a hydrocarbon network which has hydrophobic and hydrophilic regions and a balance which is variable and explains the differences in their biological properties including choleretic potency, solubilization properties and activation of BAs receptors. Hydrophobic BAs are potent detergents whereas hydrophilic BAs are not; they lack membrane-disrupting properties, thus being non-hepatotoxic, even in high concentrations. This is relevant for the therapeutic use of hydrophilic BAs, such as ursodeoxycholic acid (UDCA), tauroursodeoxycholic acid (TUDCA) and new semisynthetic BAs derivatives such as 24-norursodeoxycholic acid (Nor-UDCA) in the treatment of liver diseases [12]. Hydrophilic BAs may enhance alkalinization of bile by increasing bicarbonate secretion, therefore protecting cholangiocytes from bile cytotoxic effects. In addition, hydrophilic BAs bind in the intestines and bile ducts to nuclear and surface receptors such as FXR, TGR5 and Pregnane X receptor (PXR) to modulate inflammatory pathways [1,9].

### 2.5. Profibrotic State

Ultimately, proliferation of cholangiocytes can lead to disruption of cell cycle leading to biliary fibrosis. By secreting cytokines and chemokines via β-catenin and higher levels of intracellular cAMP, cholangiocytes attract macrophages that in turn active myofibroblasts and the transforming growth factor beta (TGFβ) [3,13]. A proinflammatory local state characterized by senescence and apoptosis may lead to ductopenia and increase other proliferative factors leading to local angiogenesis and fibrosis. At same time, there is recruitment adaptative and innate immune cells that may active mesenchymal cells and endothelial cells (Figure 2) [13].

## 3. Therapeutic Options

Based on pathophysiology, the treatment of cholestasis should pursue the following objectives: limit BAs injury by modulating their hydrophobicity or reduce the size of the pool by interfering with the intestinal absorption, induce choleresis to deload hepatocytes from BAs, limit cholangiocytes damage and modulate inflammation by reducing immune cell recruitment and activity.

### 3.1. Hydrophilic Bile Acids (BA): UDCA and Nor-UDCA

UDCA (3α,7β-dihydroxy-5β-cholanoic acid) accounts for 1–3% of human bile, and most of it is conjugated with glycine. When administered orally, it turns the BAs pool more hydrophilic (up to 40%), thus reducing the toxic effect of hydrophobic BAs [12]. This has been shown in patients with PSC, reaching a plateau at doses of 22–25 mg/kg [14]. Although a previous study showed an unchanged hydrophobic BAs pool [15], the discrepancies in the evidence led to a deeper investigation regarding the effects of UDCA in CLD. Since then, many other mechanisms of action have been described as an intracellular signaling molecule (Ca^2+^ agonist), activator of protein kinases (MAPK: Erk1/2, p38MAPK) and α5β1 integrins in hepatocytes, and a signaling molecule that stimulates vesicular exocytosis and, therefore, causes a choleretic effect. There is also the “The biliary HCO^3−^ umbrella” hypothesis which states that the alkaline pH near the apical surface due to HCO^3−^ secretion by hepatocytes and cholangiocytes prevents the permeation of hydrophobic BAs (Figure 1) [16].

The clinical effect of UDCA was first proven in patients with PBC in a prospective study that showed improvement in liver function tests and in pruritus [17]. Later, it was shown in placebo-controlled trials that it improves liver histology and delays progression to cirrhosis and LT [18,19,20]. Because of its efficacy and safety, UDCA is recommended for all patients with PBC as a first-line therapy [21].

In patients with PSC the use of UDCA as treatment has not shown such positive results. A 2009 meta-analysis of randomized controlled trials showed that UDCA can improve liver biochemistry but has no effect on liver histology or survival free of transplantation [22]. Another meta-analysis comprising 567 patients found no significant difference in mortality [OR, 0.6 (95% CI, 0.4–1.4)], main symptoms such as pruritus [OR, 1.5 (95% CI, 0.3–7.2)], or fatigue [OR, 0.0 (95% CI, 0.1–7.7)], and no difference in the risk of cholangiocarcinoma [OR, 1.7 (95% CI, 0.6–5.1)] and in histology stage progression [OR, 0.9 (95% CI, 0.34–2.44)] [23]. A 5-year Scandinavian multicenter trial with high UDCA doses (17–23 mg/kg/d) vs. placebo could not show a significant difference in symptoms, liver biochemistry, risk of cholangiocarcinoma, death, or LT. [24]. Studies about testing higher UDCA doses (28–30 mg/kg/d) had to be stopped prematurely because of severe adverse events [25]. There is scant evidence about the worsening of liver biochemistry when UDCA is discontinued in patients with PSC. In this regard, one study showed improvement when therapy was reinstated [26].

UDCA, in patients with PSC, has also been studied as chemoprophylaxis for colorectal cancer and cholangiocarcinoma, but data was conflicted. Some small studies (52 and 59 patients) have shown a lower incidence of colorectal dysplasia in patients with PSC treated with UDCA [27,28]. A randomized clinical trial of 98 patients with UDCA 17–23 mg/kg/d, showed no difference in the rate of colorectal neoplasia at a 5-year follow-up or a 15-year follow-up [29], and higher doses (28–30 mg/kg/d) have been associated with more risk of low-grade colonic dysplasia [30]. One meta-analysis found a protective effect of UDCA for colorectal cancer and/or high-grade dysplasia, which did not hold for early lesions or for overall risk [31]. Another meta-analysis showed no significant effect of UDCA on risk of colorectal neoplasia [32].

Due to the shown evidence, UDCA is the first-line therapy in PBC and PSC according to EASL and AASLD. In PSC patients, UDCA can be used at doses of 13–23 mg/kg/d, because of its effect in liver biochemistry [21,33].

Regarding Nor-UDCA, it undergoes cholehepatic shunting, resulting in ductular targeting, bicarbonate-rich hypercholeresis and cholangiocyte protection. Moreover, it can suppress the activation of pro-inflammatory signaling pathways, such as NF-κB and STAT3, thereby reducing the production of pro-inflammatory cytokines and chemokines. Additionally, Nor-UDCA can inhibit the migration and activation of immune cells involved in the inflammatory response in PSC, such as T cells and macrophages. In addition, Nor-UDCA plays a role in regulating bile acid homeostasis in PSC. It can affect the expression and activity of various transporters involved in bile acid uptake, efflux and detoxification. By modulating these transporters, Nor-UDCA helps restore bile acid balance and reduce cholestasis in PSC [12]. In a study by Fickert and colleagues, 116 patients with PSC without UDCA treatment were enrolled to receive Nor-UDCA or placebo for a 12-week and then a 4-week follow-up. The study concluded that Nor-UDCA improves ALP levels with a good safety profile [34]. Currently, there are studies exploring the effects of Nor-UDCA with promising results, especially because its efficacy in immunometabolism [12].

### 3.2. FXR Agonists: Obeticholic Acid, Tropifexor (LJN-452), Cilofexor (GS-9674) and EDP-305

This group of molecules are compounds that lack the classical BAs structure, but are able to bind and activate FXR, inducing beneficial effects such as reduction in hepatic fibrosis. This pharmaceutical approach is potentially used in the same way as the second therapeutic line in patients with an inadequate response to UDCA (approximately 40% of PBC) [11,35].

The most prominent FXR agonist is Obeticholic acid (OCA), a potent FXR agonist approved for the treatment of PBC that has had its safety and efficacy demonstrated. According to the guidelines, OCA treatment should be considered in patients with PBC who have an inadequate response to or are intolerant to UDCA. The recommended starting dosage of OCA is 5 mg once daily, with a potential increase to 10 mg after 3 months if tolerated and if ALP reduction is inadequate. Furthermore, liver biochemistry monitoring is crucial during OCA therapy. If a patient did not reach a reduction in ALP levels by at least 40% from baseline, treatment discontinuation should be considered. Although OCA has demonstrated potential benefits in improving liver biochemistry, there is currently no definitive evidence supporting its impact on long-term clinical outcomes, such as liver transplantation or mortality and like many drugs its effects in patients with cirrhosis remain uncertain [21].

For the case of PSC, a meta-analysis revealed that OCA treatment resulted in a significant reduction in ALP levels, bilirubin levels and liver stiffness, indicating improvements in liver function and fibrosis [36]. Current guidelines consider OCA therapy for patients with PSC who have ALP levels greater than 1.5 times the upper limit of normal levels despite optimal management. On the other hand, although evidence of combination therapy is limited, the use of OCA and other agents, such as fibrates or immunosuppressive drugs, may be considered in selected patients [33,36].

Tropifexor is a non-bile FXR agonist that has been shown to be effective in reducing liver fibrosis. In animal models, tropifexor upregulates FXR target genes, BSEP and small heterodimer partner (SHP) while downregulating CYP8B1 activity. This drug is currently approved for the treatment of non-alcoholic steatohepatitis (NASH) and cholestasis. It seems to be superior to OCA for the treatment of NASH and primary bile acid diarrhea [37].

In a phase 2 clinical trial by Schramm et al., tropifexor was generally well tolerated by patients with PBC who received daily doses of 30 μg, 60 μg or 90 μg for a month. There were not significant adverse events reported during the analysis period and it improved levels of markers of bile duct injury at very low doses. The primary endpoint of the study was the reduction in serum alkaline phosphatase (ALP) levels. Secondary endpoints include changes in other liver biochemistry markers, histological improvement and safety assessments. Patients who received tropifexor experienced a significant reduction in serum ALP levels compared to baseline, indicating a potential improvement in liver function. There was also a reduction of HDL by 33% at doses of 60 μg and 26% in the 90 μg group [33,38].

In a more recent study, 61 enrolled patients received 30, 60, 90 and 150 μg of tropifexor, respectively, whereas other 21 patients received a placebo. By day 28, tropifexor caused 26–72% reduction in γ-glutamyltransferase (GGT) from baseline at 30 to 150 μg doses (*p* < 0.001 at 60, 90 and 150 μg tropifexor vs. placebo). Pruritus was the most frequent adverse event in tropifexor group with most events being of mild-to-moderate severity. Decreases were also observed in LDL, HDL, and total cholesterol levels. Researchers conclude that tropifexor showed an improvement in cholestatic markers when compared to placebo [37].

Cilofexor is another FXR agonist tested in a phase 2 double-blind, placebo-controlled study in patients with PSC and without liver cirrhosis. They were randomized to receive cilofexor 100 mg, 30 mg or placebo orally once daily for 12 weeks. A total of 52 patients were randomized by dose-dependent reductions. In the group of 100 mgs, it was observed that at week 12, a significant reduction in serum ALP (median reduction −21%; *p* = 0.029 versus placebo), GGT (−30%; *p* < 0.001), alanine aminotransferase (ALT) (−49%; *p* = 0.009) and aspartate aminotransferase (AST) (−42%; *p* = 0.019). Adverse events were similar between cilofexor and placebo-treated patients; the more frequent adverse effect was pruritus, particularly in patients treated with the higher dose [36]. In a more recent study, Trauner et al. assessed the long-term effects of cilofexor in PSC patients who had previously participated in a phase 2 trial and demonstrated a positive response to the treatment. The study enrolled a cohort of PSC patients who received cilofexor once daily for 96 weeks in an open-label extension phase. Patients experienced sustained reductions in ALP levels, but also in AST, ALP and GGT. Furthermore, histological assessments showed a stabilization or improvement in liver fibrosis, suggesting the potential for cilofexor to modify the disease course [39]. Those findings led to continuation in exploring the clinical impact of cilofexor. Currently the PRIMIS trial is ongoing and is the largest randomized study about the use of cilofexor in PSC.

Finally, in the INTREPID study, 68 patients with PBC were randomized to 12-week treatment with EDP-305, a potent non-bile acid FXR agonist, which has been shown to suppress liver injury and fibrosis in animal models. Participants were randomized to receive either EDP-305 at a daily dose of 2.5 mg or 5 mg, or a placebo for a 52-week treatment period. Significant reductions in ALP levels compared to placebo particularly at 5 mg dose were found. Moreover, EDP-305 treatment was associated with favorable changes in GGT and bilirubin levels. Histological assessments showed a trend towards histological improvement in the EDP-305 group, although statistical significance was not reached [40]. On the other hand, EDP-305 may have other beneficial effects such as in bone health since it targets estrogen receptors, acting as an agonist in bone tissue to maintain or enhance bone density, potentially addressing conditions such as osteoporosis which is a long-term complication in patients with CLD. However, this effect is not currently under clinical investigation.

### 3.3. Fibroblast Growth Factor 19 (FGF-19) Analogs

NGM282 (also known as M70) is the first engineered non-tumorigenic endocrine hormone FGF19 analogue. Its primary target is to modulate the expression and activity of CYP7A1 since it is responsible for the conversion of cholesterol into primary BAs. The inhibition of CYP7A1 by NGM282 ultimately results in decreased production of BAs in the liver [10]. In animal models of PSC, treatment with NGM282 resulted in a rapid and robust reduction in ALP, ALT and AST concentrations and improvement in histological features associated with PSC.

To explore the latter effects, a multicenter, randomized, double-blind, placebo-controlled phase 2 study evaluated the efficacy and safety of NGM282 vs. the placebo for 12 weeks in patients with PSC. The study included 62 patients with PSC who were randomly assigned to receive 1 or 3 mg of NGM282 or a placebo once daily. Despite the fact that treatment with NGM282 led to improvements in ALP and GGT levels, there were no significant differences in the mean change from baseline in ALP between the intervention and placebo groups. Adverse events were mainly gastrointestinal symptoms that were more frequent in the treatment groups [10]. Another double-blind, placebo-controlled phase 2 trial, but in patients with PBC who had an inadequate response to UDCA, was carried out for 28 days. It included 45 PBC patients who were randomly assigned to receive subcutaneous doses of NGM282 at 0.3 mg, 3 mg, or a placebo daily. At the end of the study, about half of the patients who received 0.3 mg of NGM282 and 46% of those receiving 3 mg achieved a reduction in ALP levels from baseline, whereas only 7% of patients receiving the placebo achieved such a reduction [41].

To extend the effects of FGF-19 analogs, there is certain evidence relating to the combination of NGM282 and Aldafermin, another engineered FGF-19 analog, in terms of safety and tolerability in healthy volunteers and in patients with NASH. This evidence shows rapid and significant reductions in liver fat content with an acceptable safety profile [42,43].

### 3.4. PPAR Agonists: Fibrates, Seladelpar, Elafibranor

Peroxisome proliferator-activated receptors (PPARs) are nuclear receptors involved in the regulation of metabolic homeostasis. There are three main PPARs: PPARα that mainly influences fatty acid metabolism; PPARβ/δ that participates in fatty acid oxidation and regulates blood glucose and cholesterol levels; and PPARγ that regulates adipogenesis, energy balance, and lipid biosynthesis. They have therefore been studied and used for a long time as therapeutic targets in metabolic diseases; for example, thiazolidinediones in diabetes or fibrates in hypertriglyceridemia [44,45]. Furthermore, fenofibrate a PPARα agonist which is highly expressed in muscle, heart and liver is used to treat patients with CLD who are refractory to UDCA monotherapy. When activated, PPARα downregulates BAs synthesis through inhibition of the BAs-synthesizing enzymes, cytochrome P450, cholesterol 7A1-hydroxylase (CYP7A1) and cytochrome sterol 27-hydroxylase (CYP27A1). Moreover, ligands of PPARα may play a role in the regulation of bile excretory function trough up-regulation human MDR3 expression, which is localized to the canalicular membrane of hepatocytes, where it is the major determinant of biliary salts secretion, thereby reducing cholestasis [46].

Despite several trials, there is insufficient data to establish the safety and efficacy of fibrates on PBC. To date, most studies involve the combination of UDCA and bezafibrate (400 mg daily) or fenofibrate (150–200 mg daily). The first studies about the use of bezafibrate found an improvement in ALP, GGT and IgM levels but also pruritus [46,47,48,49,50,51,52]. A study by Ohmoto et al. reported an improvement in serum markers of hepatic fibrosis (7S domain of type IV collagen, the triple-helix do-main of type IV collagen, hyaluronic acid and procollagen type III N-terminal propeptide) [53]. On the other hand, since few studies have addressed the fibrotic effects of fibrates through histological assessment, such effects remain unclear.

Regarding the use of fenofibrate, at least 6 trials were conducted in the first decade of 2000s, showing an improvement in transaminases and IgM levels [52,53,54,55,56,57,58]. In this matter, a metanalysis reported that the combined therapy of UDCA and fenofibrate was better in reducing ALP than UDCA monotherapy, but not in improving clinical symptoms [59].

More recently, the BEZURSO trial showed a 60% reduction in ALP levels after only 3 months of add-on bezafibrate therapy to UDCA [60], whereas another trial in 2021 conclude that combined therapy of UDCA plus bezafibrate was associated with a significant decrease in all-cause and liver-related mortality or need for LT [61].

Another novel PPARδ agonist is MBX-8025 also known as seladelpar. The first study on its efficacy was published in 2017. It was a double-blind and placebo-controlled trial that included 70 patients. Patients were randomly assigned to receive a placebo or seladelpar at low and high doses for 12 weeks while UDCA was continued. Despite seladelpar normalizing ALP levels after 12 weeks of treatment in both groups (50 mg/day, or 200 mg/day), the study was stopped early because it was associated with increase in aminotransferases levels [62].

The ENHANCE trial on safety and efficacy of seladelpar in patients with PBC included 112 patients unresponsive to UDCA. They received seladelpar at doses of 2 mg, 5 mg and 10 mg for 1 year. The dosage was increased up to 10 mg after 12 weeks, depending on the biochemical response. In phase 3, the investigators reported an absolute reduction in ALP of nearly 45% with the 10 mg dose, which is a decrease of approximately 122 units. Other serum liver tests reflected a similar benefit from seladelpar. Adverse events were mild to moderate, and the most common problem was pruritus [63]. More recently, an open-label study in patients with PBC evaluated the effects of 1 year of seladelpar treatment on quality life and pruritus. It was carried out by self-reported experiences of 101 patients with PBC using the pruritus visual analog scale (VAS), 5D-itch scale and PBC-40 questionnaires along with BAs profiles. They received a daily dose of 5 and 10 mg of seladelpar. After 1 year of treatment, the patients improved pruritus and therefore sleep disturbances and had significant reductions in serum BAs [64].

The therapeutic effect of seladelpar relies on downregulation of BAs synthesis and modulation in their transport and metabolism. Studies in rodents reported that seladelpar reduces hepatocyte CYP7A1 via the fibroblast growth factor 21 signaling pathway. CYP7A1 is a rate-limiting enzyme in the classic pathway of BAs synthesis [65,66].

Whether seladelpar should be used in the first-line or as an add-on to UDCA for patients with PBC should be assessed. Several clinical trials assessing either efficacy or safety of seladelpar are currently ongoing.

Another PPAR agonist is elafibranor which initially emerged as therapy for NASH due to its effects over metabolic syndrome and expression of proinflammatory genes that may result in antifibrotic properties [67]. In regard to cholestasis, elafibranor activates PPARα which inhibits CYP7A1 expression leading to a reduction in BAs synthesis, whereas the induction of CYP3A4, SULT2A1 and UGT2B4, and the expression of BSEP and MRP2, decrease BAs output and toxicity [68]. Moreover, in the same spectrum of its antifibrotic effects, elafibranor may have beneficial effects in the progression of PBC and PSC. The reduction in fibrosis is achieved by the activation of PPARα and PPARδ that lead to the inhibition of NF-κB and AP-1 pathways. These two pathways are related to liver damage and inflammation [69,70,71].

A phase 2 clinical trial about the effects of elafibranor in patients with PBC was carried out during a 12-week administration of 80 mg of elafibranor in 45 patients with PBC and incomplete response to UDCA. The study found that the intervention reduced the levels of ALP, total bilirubin and inflammatory markers such as C-reactive protein. There was no effect on pruritus [67]. Despite the promising molecular targets of elafibranor, there is still insufficient information about the efficacy and safety of this drug. In 2020, phase 2 of ELATIVE clinical trial about the efficacy and safety of elafibranor in patients with PBC and inadequate response or intolerance to UDCA was announced [68].

### 3.5. ASBT Inhibitors

About 90–95% of BAs are reabsorbed into the terminal ileum via ASBT. Current evidence explains that the inhibition of ASBT reduces the overload of BAs in the liver. The decrease in ASBT expression increases the excretion of fecal BAs and the total concentration of BAs in liver [72]. On the other hand, the interruption of BAs enterohepatic circulation which lowers their concentrations in the liver also leads to bile salt spilling over to the colon, resulting in diarrhea, irregular bowel movement, and abdominal pain [73,74]. A recent study in mice found that inhibition of ASBT in the kidneys stimulates the renal bile salts excretion and consequently improves intestinal side effects [74]. Yet, new ASBT inhibitors such as Odevixibat, Lopixibat and GSK2330672 are restricted to intestinal ASBT blockading.

The first ASBT inhibitor approved by the FDA was Lopixibat (Maralixibat) but only for children with Alagille syndrome which causes severe cholestasis and pruritus due to deletion or mutation of the JAG1 gene and leads to defective bile duct development. The use of Maralixibat 380 μg/kg once per day and the subsequent increase to 380 μg/kg twice per day reduced serum BAS and pruritus according to a placebo-controlled study [75].

Regarding GSK2330672 (Linerixibat), in 2017, a double-blind, randomized, placebo-controlled trial with 22 patients reported that the administration of GSK2330672 for 2 weeks improved pruritus (assessed by itch scores) and total serum BAs concentrations declined by 50% from baseline after receival of the intervention. Despite intestinal adverse effects, the drug was well tolerated [76]. In this matter, a later study in healthy Japanese volunteers investigated the safety and tolerability of GSK2330672 versus a placebo. No serious adverse events were present and the main side effect was diarrhea [77]. Another, but larger, placebo-controlled trial found that GSK2330672 was not significantly different versus placebo [78].

Odevixibat is another FDA drug approved which is only limited to treat pruritus in patients aged ≥3 months with progressive familial intrahepatic cholestasis. Studies reported that this drug effectively reduces pruritus and serum BAs, and it is generally well tolerated [79,80].

### 3.6. Immune-Modulation Drugs: Corticosteroids and Biological Therapies

The use of corticosteroids in cholestasis remains an area of active investigation. In vitro studies and animal models support the beneficial effects of corticosteroids. The mechanism of these drugs in cholestasis is related with the reduction in cell edema and relieving the inflammation of the bile duct cells and liver cells. Moreover, the immunomodulatory effect over Kupffer cells and the release of reactive oxygen species (ROS) protects from liver damage and thereby improving transaminases levels and the biochemical markers of cholestasis (Figure 2) [81,82]. Clinical evidence, however, is still controversial. Two metanalyses about the combination of corticosteroids with UDCA for patients with PBC conclude that combination therapy of those two drugs did not differ significantly from the monotherapy in improving fatigue, jaundice, mortality, death/LT or adverse events, but was significantly superior to the monotherapy in reducing serum biochemical liver markers [83,84]. Most of studies on steroids in PCB are for overlap syndrome.

Budesonide is a corticosteroid that has been studied for a long time for patients with PBC because of its high glucocorticoid receptor binding affinity in the liver when compared to other steroids. It is also a PXR agonist which is involved in BAs synthesis, metabolism and transport [85]. Nevertheless, its efficacy and safety are anecdotical. The first study was randomized placebo-controlled trial; the intervention was budesonide 9 mg/day and UDCA for 2 years. Patients showed some improvement in liver histology [86]. A phase 3 trial of combining therapy with budesonide and UDCA in patients with PBC and incomplete response to UDCA failed to meet its primary endpoint of histological improvement [84]. In a more recent study, 62 patients were enrolled to receive budesonide (9 mg/day) or placebo once daily during 36 months in combination with UDCA. Unfortunately, while the addition of budesonide improves ALP levels, it did not liver histology [87]. Moreover, long-term use of budesonide is related with important side effects, especially as liver disease progresses [21,88].

Biological agents are a novel therapy for cholestasis and there still ongoing trials to evaluate their efficacy in PBC; some of those drugs are Ustekinumab or Rituximab. The latter is an anti-CD20 chimeric monoclonal antibody that is used to deplete B cells to decrease autoantibody production and antigen presentation. This is important since the hallmark of PBC is the presence of anti-mitochondrial autoantibodies and high amounts of IgM [89,90]. The same mechanism of Rituximab applies to PSC and clinical trials show beneficial effects, even for PSC recurrence after LT, though studies are still limited [90,91]. The results of two open-label studies on the efficacy of Rituximab in patients with PBC and incomplete UDCA response suggested a limited efficacy of Rituximab in PBC patients, even though an impressive reduction in ALP levels was observed [89,90].

Other biological therapies include Abatacept, a modified antibody to cytotoxic T-lymphocyte antigen 4. It was studied in an open-label trial for 24 weeks in patients with PBC but failed to demonstrate its efficacy [92].

In the case of Baricitinib, a selective JAK1 and JAK2 inhibitor, there is just one study that included two patients with PBC and inadequate response to UDCA. The patient treated with Baricitinib demonstrated a 30% decrease in ALP and improvement in itch score when compared with the placebo patient [93]. As with Baricitinib, there are other small studies of molecules such as Ustekinumab in PBC and vedolizumab in PSC patients, respectively. Unfortunately, the results of those studies failed to demonstrate the efficacy in achieving a decrease, or even moderate, in ALP levels [94,95].

Finally, the novel NI-0801 monoclonal antibody, which is an anti-CXCL10, was evaluated in a phase 2 study, enrolling 29 patients with PBC that did not respond to UDCA. After a 3-month follow-up, performed after 6 doses of NI-0801 10 mg/Kg every 2 weeks, the trial was terminated due to no significant therapeutic benefits obtained. Side effects included headaches, pruritus, fatigue and diarrhea [96]. The reason to use this molecule is that as anti-CXCL10, which secreted in response to interferon-γ-stimulation, may reduce the hepatic recruitment of inflammatory T cells [97].

Biological agents showed promising results in certain scenarios, but conflicting results have been produced and these molecules are not part of the current therapeutic options. In addition, there are few studies and a small population. More studies are required to establish long-term beneficial effects.

### 3.7. Other Therapies: Bile Acid Sequestrant and Antibiotics

The are other classic alternative therapies such as sertraline, antibiotics and BAs sequestrant. In the case of the latter group, cholestyramine is the most studied drug due to its mechanism to bind to BAs in the intestine, preventing their reabsorption and facilitating their elimination through fecal excretion.

Historically, cholestyramine is recommended as therapy for the symptomatic relief of pruritus in CLD, including PBC. It is advised to initiate therapy with cholestyramine at a dose of 4 g per day, divided into 2 equal administrations, to a maximum of 16 g per day [21,33]. The findings in a study by Tandon et al. about BAs binding agents, such as cholestyramine and colestipol, demonstrated significant efficacy in reducing pruritus associated with cholestasis. These agents were found to provide relief from itching symptoms in a majority of patients, with a statistically significant improvement compared to placebo or control groups. However, it was noted that the individual response to bile acid BAs varied, and some patients may not experience complete resolution of pruritus [98]. In contrast, other BAs sequestrants such as colesevelam are not effective in cholestatic pruritus [99].

On the other hand, antibiotics have been studied to treat bacterial proliferation in the small intestine, which is a complication of cholestatic diseases and results from the association with inflammatory bowel disease. In this matter, a metanalysis indicated that antibiotic therapy may confer some benefits in PSC patients, regardless of the presence of concomitant inflammatory bowel disease, mainly with the use of metronidazole, vancomycin and rifaximin. There was an improved liver biochemistry, including reduced serum liver enzyme levels, as well as in Mayo PSC Risk Score [100]. The results of another metanalysis also in the context of PSC were inconclusive [101].

The basis of using antibiotics in CLD is that gut microbiota changes the profile of BAs as CLD progresses, whereas at the same time, there is an intrinsic change in BAs composition due to liver fibrosis which alters gut microbiota, inducing dysbiosis and the release of bacterial pathogen-associated molecular patterns (PAMPS) into the enterohepatic circulation, hence activating immune cells in the liver [102]. Some studies identified the immunomodulating effects of oral vancomycin in the gut by reducing cytokine release from T cells, and as an antimicrobial agent [103,104]. In contrast, the use of rifampicin is still controversial; in a metanalysis rifampicin appears to be a safe and well-tolerated option for the treatment of pruritus in CLD. The study findings suggest a low incidence of adverse events associated with rifampicin therapy. Nevertheless, individual patient factors and potential drug interactions should be carefully evaluated before commencing rifampicin treatment [105]. To date, there are few new studies on the use of rifampicin in CLD. The most recent was a multi-center, open label, randomized trial published in 2021 about the superiority of rifampicin vs. UDCA in intrahepatic cholestasis of pregnancy with poor results in favor of rifampicin [106].

## 4. Conclusions

To date, there is a wide spectrum of therapeutic options for CLD, most of them for the treatment of PBC and PSC. Nevertheless, the evidence of long-term benefits is controversial even if treatments possess a good safe profile. Moreover, few therapies are currently approved; according to guidelines, UDCA is the first line of treatment in patients with PBC followed by OCA, while in patients with PSC, UDCA remains the most recommended drug to date. The latter recommendations are made since an important proportion of those patients are intolerant or unresponsive to UDCA. Moreover, although quality of life may improve with UDCA, symptoms such as pruritus decrease, liver histology and disease progression may not improve. Regarding this matter, several drugs have been tested in CLD with a variety of results (Figure 2). PPAR agonist and other FXR agonist seems to be the best options in PBC, especially when combined with UDCA. For PPAR agonist, the use of fibrates is supported by strong evidence on efficacy and safety in PBC, perhaps because their time on the market; however, they lack sufficient evidence in PSC. On the other hand, Elafibranor has interesting results for liver fibrosis. Unfortunately, there are few studies that address its use in CLD, and although it may delay disease progression, its mechanism of action may not be enough to help with symptoms such as fatigue and pruritus. FXR agonists is the most prominent group as alternative to treat PSC, followed by FGF-19 analogs and cilofexor, which have shown promising results that lead to the development of more clinical trials. In the same matter of PSC, biological therapies and steroids have been studied, especially because of PSC association with inflammatory bowel disease. However, although the effectiveness of targeting these mechanisms has been observed in theory and in vitro models, its efficacy in clinical trials has not been established. In the same scenario, antibiotics may offer assistance, but as a concomitant treatment.

The case of ASBT inhibitors is a good example of success, they have a good safety profile despite intestinal side effects and have FDA approbation. Yet, there is insufficient evidence to use in other CLD besides Alagille syndrome. In conclusion, it seems that UDCA, OCA and even Nor-UDCA will remain as main therapies for PBC and PSC, but the addition of FXR agonist and PPAR agonist may improve the management of liver cirrhosis.

## References

[1] Sanjel B., Shim W.S. (2020). Recent advances in understanding the molecular mechanisms of cholestatic pruritus: A review. Biochim. Biophys. Acta Mol. Basis.

[2] Gazda J., Drazilova S., Janicko M., Jarcuska P. (2021). The Epidemiology of Primary Biliary Cholangitis in European Countries: A Systematic Review and Meta-Analysis. Can. J. Gastroenterol. Hepatol..

[3] Tabibian J.H., Ali A.H., Lindor K.D. (2018). Primary Sclerosing Cholangitis, Part 1: Epidemiology, Etiopathogenesis, Clinical Features, and Treatment. Gastroenterol. Hepatol..

[4] Malik A., Kardashian A.A., Zakharia K., Bowlus C.L., Tabibian J.H. (2019). Preventative care in cholestatic liver disease: Pearls for the specialist and subspecialist. Liver Res..

[5] Gerussi A., Restelli U., Croce D., Bonfanti M., Invernizzi P., Carbone M. (2021). Cost of illness of Primary Biliary Cholangitis—A population-based study. Dig. Liver Dis..

[6] Yokoda R.T., Rodriguez E.A. (2020). Review: Pathogenesis of cholestatic liver diseases. World J. Hepatol..

[7] Méndez-Sánchez N. (2017). Bile Acids in Health and Disease Foreword. Ann. Hepatol..

[8] Desmet V.J. (2011). Ductal plates in hepatic ductular reactions. Hypothesis and implications. I. Types of ductular reaction reconsidered. Virchows Arch..

[9] Goldstein J., Levy C. (2018). Novel and emerging therapies for cholestatic liver diseases. Liver Int..

[10] Hirschfield G.M., Chazouillères O., Drenth J.P., Thorburn D., Harrison S.A., Landis C.S., Mayo M.J., Muir A.J., Trotter J.F., Leeming D.J. (2019). Effect of NGM282, an FGF19 analogue, in primary sclerosing cholangitis: A multicenter, randomized, double-blind, placebo-controlled phase 2 trial. J. Hepatol..

[11] Méndez-Sánchez N. (2018). Management of primary biliary cholangitis: The importance to identify patients’ non-responders to standard treatment. Minerva Med..

[12] Cabrera D., Arab J.P., Arrese M. (2019). UDCA, NorUDCA, and TUDCA in Liver Diseases: A Review of Their Mechanisms of Action and Clinical Applications. Handb. Exp. Pharmacol..

[13] Fabris L., Fiorotto R., Spirli C., Cadamuro M., Mariotti V., Perugorria M.J., Banales J.M., Strazzabosco M. (2019). Pathobiology of inherited biliary diseases: A roadmap to understand acquired liver diseases. Nat. Rev. Gastroenterol. Hepatol..

[14] Hartl L., Haslinger K., Angerer M., Semmler G., Schneeweiss-Gleixner M., Jachs M., Simbrunner B., Bauer D.J.M., Eigenbauer E., Strassl R. (2022). Progressive cholestasis and associated sclerosing cholangitis are frequent complications of COVID-19 in patients with chronic liver disease. Hepatology.

[15] Beuers U., Spengler U., Kruis W., Aydemir U., Wiebecke B., Heldwein W., Weinzierl M., Pape G.R., Sauerbruch T., Paumgartner G. (1992). Ursodeoxycholic acid for treatment of primary sclerosing cholangitis: A placebo-controlled trial. Hepatology.

[16] Beuers U., Trauner M., Jansen P., Poupon R. (2015). New paradigms in the treatment of hepatic cholestasis: From UDCA to FXR, PXR and beyond. J. Hepatol..

[17] Poupon R., Chrétien Y., Poupon R.E., Ballet F., Calmus Y., Darnis F. (1987). Is ursodeoxycholic acid an effective treatment for primary biliary cirrhosis?. Lancet.

[18] Poupon R.E., Balkau B., Eschwège E., Poupon R. (1991). A multicenter, controlled trial of ursodiol for the treatment of primary biliary cirrhosis. UDCA-PBC Study Group. N. Engl. J. Med..

[19] Poupon R.E., Balkau B., Guéchot J., Heintzmann F. (1994). Predictive factors in ursodeoxycholic acid-treated patients with primary biliary cirrhosis: Role of serum markers of connective tissue. Hepatology.

[20] Poupon R.E., Bonnand A.M., Chrétien Y., Poupon R. (1999). Ten-year survival in ursodeoxycholic acid-treated patients with primary biliary cirrhosis. UDCA-PBC Study Group Hepatol..

[21] European Association for the Study of the Liver (2017). EASL Clinical Practice Guidelines: The diagnosis and management of patients with primary biliary cholangitis. J. Hepatol..

[22] Shi J., Li Z., Zeng X., Lin Y., Xie W.F. (2009). Ursodeoxycholic acid in primary sclerosing cholangitis: Meta-analysis of randomized controlled trials. Hepatol. Res..

[23] Triantos C.K., Koukias N.M., Nikolopoulou V.N., Burroughs A.K. (2011). Meta-analysis: Ursodeoxycholic acid for primary sclerosing cholangitis. Aliment. Pharmacol. Ther..

[24] Olsson R., Boberg K.M., de Muckadell O.S., Lindgren S., Hultcrantz R., Folvik G., Bell H., Gangsøy-Kristiansen M., Matre J., Rydning A. (2005). High-dose ursodeoxycholic acid in primary sclerosing cholangitis: A 5-year multicenter, randomized, controlled study. Gastroenterology.

[25] Lindor K.D., Kowdley K.V., Luketic V.A., Harrison M.E., McCashland T., Befeler A.S., Harnois D., Jorgensen R., Petz J., Keach J. (2009). High-dose ursodeoxycholic acid for the treatment of primary sclerosing cholangitis. Hepatology.

[26] Black D.D., Mack C., Kerkar N., Miloh T., Sundaram S.S., Anand R., Gupta A., Alonso E., Arnon R., Bulut P. (2019). A Prospective Trial of Withdrawal and Reinstitution of Ursodeoxycholic Acid in Pediatric Primary Sclerosing Cholangitis. Hepatol. Commun..

[27] Pardi D.S., Loftus E.V., Kremers W.K., Keach J., Lindor K.D. (2003). Ursodeoxycholic acid as a chemopreventive agent in patients with ulcerative colitis and primary sclerosing cholangitis. Gastroenterology.

[28] Tung B.Y., Emond M.J., Haggitt R.C., Bronner M.P., Kimmey M.B., Kowdley K.V., Brentnall T.A. (2001). Ursodiol use is associated with lower prevalence of colonic neoplasia in patients with ulcerative colitis and primary sclerosing cholangitis. Ann. Intern. Med..

[29] Lindström L., Boberg K.M., Wikman O., Friis-Liby I., Hultcrantz R., Prytz H., Sandberg-Gertzén H., Sangfelt P., Rydning A., Folvik G. (2012). High dose ursodeoxycholic acid in primary sclerosing cholangitis does not prevent colorectal neoplasia. Aliment. Pharmacol. Ther..

[30] Eaton J.E., Silveira M.G., Pardi D.S., Sinakosm E., Kowdley K.V., Luketic V.A., Harrison M.E., McCashland T., Befeler A.S., Harnois D. (2011). High-dose ursodeoxycholic acid is associated with the development of colorectal neoplasia in patients with ulcerative colitis and primary sclerosing cholangitis. Am. J. Gastroenterol..

[31] Singh S., Khanna S., Pardi D.S., Loftus E.V., Talwalkar J.A. (2013). Effect of ursodeoxycholic acid use on the risk of colorectal neoplasia in patients with primary sclerosing cholangitis and inflammatory bowel disease: A systematic review and meta-analysis. Inflamm. Bowel Dis..

[32] Hansen J.D., Kumar S., Lo W.K., Poulsen D.M., Halai U.A., Tater K.C. (2013). Ursodiol and colorectal cancer or dysplasia risk in primary sclerosing cholangitis and inflammatory bowel disease: A meta-analysis. Dig. Dis. Sci..

[33] Bowlus C.L., Arrivé L., Bergquist A., Deneau M., Forman L., Ilyas S.I., Lunsford K.E., Martinez M., Sapisochin G., Shroff R. (2023). AASLD practice guidance on primary sclerosing cholangitis and cholangiocarcinoma. Hepatology.

[34] Fickert P., Hirschfield G.M., Denk G., Marschall H.U., Altorjay I., Färkkilä M., Schramm C., Spengler U., Chapman R., Bergquist A. (2017). norUrsodeoxycholic acid improves cholestasis in primary sclerosing cholangitis. J. Hepatol..

[35] Floreani A., Gabbia D., de Martin S. (2022). Update on the Pharmacological Treatment of Primary Biliary Cholangitis. Biomedicines.

[36] Smith T., Dunkelberg J., Roy P.K. (2012). Obeticholic acid for the treatment of primary sclerosing cholangitis: A systematic review and meta-analysis. Arab. J. Gastroenterol..

[37] Schramm C., Wedemeyer H., Mason A., Hirschfield G.M., Levy C., Kowdley K.V., Milkiewicz P., Janczewska E., Malova E.S., Sanni J. (2022). Farnesoid X receptor agonist tropifexor attenuates cholestasis in a randomised trial in patients with primary biliary cholangitis. JHEP Rep..

[38] Schramm C., Hirschfield G.M., Mason A., Wedemeyer H., Klickstein L., Neelakantham S., Koo P., Sanni J., Badman M., Jones D. (2018). Early assessment of safety and efficacy of tropifexor, a potent non bile-acid FXR agonist, in patients with primary biliary cholangitis: An interim analysis of an ongoing phase 2 study. J. Hepatol..

[39] Trauner M., Gulamhusein A., Hameed B., Caldwell S., Shiffman M.L., Landis C., Eksteen B., Agarwal K., Muir A., Rushbrook S. (2019). The Nonsteroidal Farnesoid X Receptor Agonist Cilofexor (GS-9674) Improves Markers of Cholestasis and Liver Injury in Patients with Primary Sclerosing Cholangitis. Hepatology.

[40] Trauner M., Bowlus C.L., Gulamhusein A., Hameed B., Caldwell S.H., Shiffman M.L., Landis C., Muir A.J., Billin A., Xu J. (2023). Safety and sustained efficacy of the farnesoid X receptor (FXR) agonist cilofexor over a 96-week open-label extension in patients with PSC. Clin. Gastroenterol. Hepatol..

[41] Kowdley K.V., Bonder A., Heneghan M.A., Hodge A.D., Ryder S.D., Sanchez A.J., Vargas V., Zeuzem S., Ahmad A., Larson K. (2020). Final data of the phase 2a intrepid study with EDP-305, a non-bile acid farnesoid x receptor (FXR) agonist. Hepatology.

[42] Mayo M.J., Wigg A.J., Leggett B.A., Arnold H., Thompson A.J., Weltman M., Carey E.J., Muir A.J., Ling L., Rossi S.J. (2018). NGM282 for Treatment of Patients with Primary Biliary Cholangitis: A Multicenter, Randomized, Double-Blind, Placebo-Controlled Trial. Hepatol. Commun..

[43] Harrison S.A., Rinella M.E., Abdelmalek M.F., Trotter J.F., Paredes A.H., Arnold H.L., Kugelmas M., Bashir M.R., Jaros M.J., Ling L. (2018). NGM282 for treatment of non-alcoholic steatohepatitis: A multicentre, randomised, double-blind, placebo-controlled, phase 2 trial. Lancet.

[44] Harrison S.A., Neff G., Guy C.D., Bashir M.R., Paredes A.H., Frias J.P., Younes Z., Trotter J.F., Gunn N.T., Moussa S.E. (2021). Efficacy and Safety of Aldafermin, an Engineered FGF19 Analog, in a Randomized, Double-Blind, Placebo-Controlled Trial of Patients with Nonalcoholic Steatohepatitis. Gastroenterology.

[45] Botta M., Audano M., Sahebkar A., Sirtori C.R., Mitro N., Ruscica M. (2018). PPAR Agonists and Metabolic Syndrome: An Established Role?. Int. J. Mol. Sci..

[46] Grygiel-Górniak B. (2014). Peroxisome proliferator-activated receptors and their ligands: Nutritional and clinical implications—A review. Nutr. J..

[47] Ghonem N.S., Assis D.N., Boyer J.L. (2015). On Fibrates and Cholestasis: A review. Hepatology.

[48] Kita R., Takamatsu S., Kimura T., Kokuryu H., Osaki Y., Tomono N. (2006). Bezafibrate may attenuate biliary damage associated with chronic liver diseases accompanied by high serum biliary enzyme levels. J. Gastroenterol..

[49] Hazzan R., Tur-Kaspa R. (2010). Bezafibrate treatment of primary biliary cirrhosis following incomplete response to ursodeoxycholic acid. J. Clin. Gastroenterol..

[50] Takeuchi Y., Ikeda F., Fujioka S., Takaki T., Osawa T., Yasunaka T., Miyake Y., Takaki A., Iwasaki Y., Kobashi H. (2011). Additive improvement induced by bezafibrate in patients with primary biliary cirrhosis showing refractory response to ursodeoxycholic acid. J. Gastroenterol. Hepatol..

[51] Iwasaki S., Ohira H., Nishiguchi S., Zeniya M., Kaneko S., Onji M., Ishibashi H., Sakaida I., Kuriyama S., Ichida T. (2008). The efficacy of ursodeoxycholic acid and bezafibrate combination therapy for primary biliary cirrhosis: A prospective, multicenter study. Hepatol. Res..

[52] Ohmoto K., Yoshioka N., Yamamoto S. (2006). Long-term effect of bezafibrate on parameters of hepatic fibrosis in primary biliary cirrhosis. J. Gastroenterol..

[53] Ohira H., Sato Y., Ueno T., Sata M. (2002). Fenofibrate treatment in patients with primary biliary cirrhosis. Am. J. Gastroenterol..

[54] Dohmen K., Mizuta T., Nakamuta M., Shimohashi N., Ishibashi H., Yamamoto K. (2004). Fenofibrate for patients with asymptomatic primary biliary cirrhosis. World J. Gastroenterol..

[55] Levy C., Peter J.A., Nelson D.R., Keach J., Petz J., Cabrera R., Clark V., Firpi R.J., Morelli G., Soldevila-Pico C. (2011). Pilot study: Fenofibrate for patients with primary biliary cirrhosis and an incomplete response to ursodeoxycholic acid. Aliment. Pharmacol. Ther..

[56] Han X.F., Wang Q.X., Liu Y., You Z.R., Bian Z.L., Qiu D.K., Ma X. (2012). Efficacy of fenofibrate in Chinese patients with primary biliary cirrhosis partially responding to ursodeoxycholic acid therapy. J. Dig. Dis..

[57] Liberopoulos E.N., Florentin M., Elisaf M.S., Mikhailidis D., Tsianos E. (2010). Fenofibrate in primary biliary cirrhosis: A pilot study. Open Cardiovasc. Med. J..

[58] Zhang H., Li S., Feng Y., Zhang Q., Xie B. (2022). Efficacy of fibrates in the treatment of primary biliary cholangitis: A meta-analysis. Clin. Exp. Med..

[59] Zhang Y., Li S., He L., Wang F., Chen K., Li J., Liu T., Zheng Y., Lu W., Zhou Y. (2015). Combination therapy of fenofibrate and ursodeoxycholic acid in patients with pri mary biliary cirrhosis who respond incompletely to UDCA monotherapy: A meta-analysis. Drug Des. Devel Ther..

[60] Corpechot C., Chazouillères O., Rousseau A., Le Gruyer A., Habersetzer F., Mathurin P., Goria O., Potier P., Minello A., Silvain C. (2018). A Placebo-Controlled Trial of Bezafibrate in Primary Biliary Cholangitis. N. Engl. J. Med..

[61] Tanaka A., Hirohara J., Nakano T., Matsumoto K., Chazouillères O., Takikawa H., Hansen B.E., Carrat F., Corpechot C. (2021). Association of bezafibrate with transplant-free survival in patients with primary biliary cholangitis. J. Hepatol..

[62] Jones D., Boudes P.F., Sawin M.G., Bowlus C.L., Galambos M.R., Bacon B.R., Doerffel Y., Gitlin N., Gordon S.C., Odin J.A. (2017). Seladelpar (MBX-8025), a selective PPAR-δ agonist, in patients with primary biliary cholangitis with an inadequate response to ursodeoxycholic acid: A double-blind, randomised, placebo-controlled, phase 2, proof-of-concept study. Lancet Gastroenterol. Hepatol..

[63] Bowlus C.L., Galambos M.R., Aspinall R.J., Hirschfield G.M., Jones D.E.J., Dörffel Y., Gordon S.C., Harrison S.A., Kremer A.E., Mayo M.J. (2022). A phase 2, randomized, open-label, 52-week study of seladelpar in patients with primary biliary cholangitis. J. Hepatol..

[64] Kremer A.E., Mayo M.J., Hirschfield G., Levy C., Bowlus C.L., Jones D.E., Steinberg A., McWherter C.A., Choi Y.J. (2022). Seladelpar improved measures of pruritus, sleep, and fatigue and decreased serum bile acids in patients with primary biliary cholangitis. Liver Int..

[65] Zhang L., Huang X., Meng Z., Dong B., Shiah S., Moore D.D., Huang W. (2009). Significance and mechanism of CYP7a1 gene regulation during the acute phase of liver regeneration. Mol. Endocrinol..

[66] Colapietro F., Gershwin M.E., Lleo A. (2023). PPAR agonists for the treatment of primary biliary cholangitis: Old and new tales. J. Transl. Autoimmun..

[67] Westerouen Van Meeteren M.J., Drenth J.P.H., Tjwa E.T.T.L. (2020). Elafibranor: A potential drug for the treatment of nonalcoholic steatohepatitis (NASH). Expert Opin. Investig. Drugs.

[68] Schattenberg J.M., Pares A., Kowdley K.V., Heneghan M.A., Caldwell S.A., Pratt D., Bonder A., Hirschfield G.M., Levy C., Vierling J. (2021). randomized placebo-controlled trial of elafibranor in patients with primary biliary cholangitis and incomplete response to UDCA. J. Hepatol..

[69] Tao L., Ren X., Zhai W., Chen Z. (2022). Progress and Prospects of Non-Canonical NF-κB Signaling Pathway in the Regulation of Liver Diseases. Molecules.

[70] Li F., Patterson A.D., Krausz K.W., Jiang C., Bi H., Sowers A.L., Cook J.A., Mitchell J.B., Gonzalez F.J. (2012). Metabolomics reveals an essential role for peroxisome proliferator-activated receptor α in bile acid homeostasis. J. Lipid Res..

[71] Ye X., Zhang T., Han H. (2022). PPARα: A potential therapeutic target of cholestasis. Front. Pharmacol..

[72] Yang N., Dong Y.Q., Jia G.X., Fan S.M., Li S.Z., Yang S.S., Li Y.B. (2020). ASBT(SLC10A2): A promising target for treatment of diseases and drug discovery. Biomed. Pharmacother..

[73] Salic K., Kleemann R., Wilkins-Port C., McNulty J., Verschuren L., Palmer M. (2019). Apical sodium-dependent bile acid transporter inhibition with volixibat improves metabolic aspects and components of non-alcoholic steatohepatitis in Ldlr-/-.Leiden mice. PLoS ONE.

[74] Kunst R.F., de Waart D.R., Wolters F., Duijst S., Vogels E.W., Bolt I., Verheij J., Beuers U., Elferink R.P.O., van de Graaf S.F. (2022). Systemic ASBT inactivation protects against liver damage in obstructive cholestasis in mice. JHEP Rep..

[75] Gonzales E., Hardikar W., Stormon M., Baker A., Hierro L., Gliwicz D., Lacaille F., Lachaux A., Sturm E., Setchell K.D.R. (2021). Efficacy and safety of maralixibat treatment in patients with Alagille syndrome and cholestatic pruritus (ICONIC): A randomised phase 2 study. Lancet.

[76] Hegade V.S., Kendrick S.F., Dobbins R.L., Miller S.R., Thompson D., Richards D., Storey J., Dukes G.E., Corrigan M., Elferink R.P.O. (2017). Effect of ileal bile acid transporter inhibitor GSK2330672 on pruritus in primary biliary cholangitis: A double-blind, randomised, placebo-controlled, crossover, phase 2a study. Lancet.

[77] Ino H., Endo A., Wakamatsu A., Ogura H., Numachi Y., Kendrick S. (2019). Safety, Tolerability, Pharmacokinetic and Pharmacodynamic Evaluations Following Single Oral Doses of GSK2330672 in Healthy Japanese Volunteer. Clin. Pharmacol. Drug. Dev..

[78] Levy C., Kendrick S., Bowlus C.L., Tanaka A., Jones D., Kremer A.E., Mayo M.J., Haque N., von Maltzahn R., Allinder M. (2022). GLIMMER: A Randomized Phase 2b Dose-Ranging Trial of Linerixibat in Primary Biliary Cholangitis Patients with Pruritus. Clin. Gastroenterol. Hepatol..

[79] Thompson R., Arnell H., Artan R., Baumann U., Calvo P.L.C., Czubkowski P., Dalgic B., D’Antiga L., Durmaz Ö., Fischler B. (2022). Odevixibat treatment in progressive familial intrahepatic cholestasis: A randomised, placebo-controlled, phase 3 trial. Lancet Gastroenterol. Hepatol..

[80] Deeks E.M. (2021). Odevixibat: First Approval. Drugs.

[81] Blendis L. (2005). Steroids in the management of PBC: Why do we need them?. Gastroenterology.

[82] Al-Aqil F.A., Monte M.J., Peleteiro-Vigil A., . Briz O., Rosales R., González R., Aranda C.J., Ocón B., Uriarte I., de Medina F.S. (2018). Interaction of glucocorticoids with FXR/FGF19/FGF21-mediated ileum-liver crosstalk. Biochim. Biophys. Acta Mol. Basis Dis..

[83] Zhang Y., Lu J., Dai W., Wang F., Shen M., Yang J., Zhu R., Zhang H., Chen K., Cheng P. (2013). Combination Therapy of Ursodeoxycholic Acid and Corticosteroids for Primary Biliary Cirrhosis with Features of Autoimmune Hepatitis: A Meta-Analysis. Gastroenterol. Res. Pract..

[84] Zhang H., Li S., Yang J., Zheng Y., Wang J., Lu W., Zhou Y., Yin Q., Zhu R., Guo C. (2015). A meta-analysis of ursodeoxycholic acid therapy versus combination therapy with corticosteroids for PBC-AIH-overlap syndrome: Evidence from 97 monotherapy and 117 combinations. Prz. Gastroenterol..

[85] Silveira M.G., Lindor K.D. (2014). Obeticholic acid and budesonide for the treatment of primary biliary cirrhosis. Expert. Opin. Pharmacother..

[86] Hempfling W., Grunhage F., Dilger K., Reichel C., Beuers U., Sauerbruch T. (2003). Pharmacokinetics and pharmacodynamic action of budesonide in early and late-stage primary biliary cirrhosis. Hepatology.

[87] Hirschfield G.M., Beuers U., Kupcinskas L., Ott P., Bergquist A., Färkkilä M., Manns M.P., Parés A., Spengler U., Stiess M. (2021). A placebo-controlled randomised trial of budesonide for PBC following an insufficient response to UDCA. J. Hepatol..

[88] Geier A., Gartung C., Dietrich C.G., Wasmuth H.E., Reinartz P., Matern S. (2003). Side effects of budesonide in liver cirrhosis due to chronic autoimmune hepatitis: Influence of hepatic metabolism versus portosystemic shunts on a patients complicated with HCC. World J. Gastroenterol..

[89] Myers R.P., Swain M.G., Lee S.S., Shaheen A.A., Burak K.W. (2013). B-cell depletion with rituximab in patients with primary biliary cirrhosis refractory to ursodeoxycholic acid. Am. J. Gastroenterol..

[90] Tsuda M., Moritoki Y., Lian Z.X., Zhang W., Yoshida K., Wakabayashi K., Yang G.X., Nakatani T., Vierling J., Lindor K. (2012). Biochemical and immunologic effects of rituximab in patients with primary biliary cirrhosis and incomplete response to ursodeoxycholic acid. Hepatology.

[91] Hart P.A., Topazian M.D., Witzig T.E., Clain J.E., Gleeson F.C., Klebig R.R., Levy M.J., Pearson R.K., Petersen B.T., Smyrk T.C. (2012). Treatment of relapsing autoimmune pancreatitis with immunomodulators and rituximab: The Mayo Clinic experience. Gut.

[92] Yamada Y., Hoshino K., Fuchimoto Y., Matsubara K., Hibi T., Yagi H., Abe Y., Shinoda M., Kitago M., Obara H. (2018). Rituximab induction to prevent the recurrence of PSC after liver transplantation—The lessons learned from ABO-Incompatible living donor liver transplantation. Transplant. Direct..

[93] Bowlus C.L., Yang G.-X., Liu C.H., Johnson C.R., Dhaliwal S.S., Frank D., Levy C., Peters M.G., Vierling J.M., Gershwin M.E. (2019). Therapeutic trials of biologics in primary biliary cholangitis: An open label study of abatacept and review of the literature. J. Autoimmun..

[94] Gordon S.C., Trudeau S., Regev A., Uhas J.M., Chakladar S., Pinto-Correia A., Gottlieb K., Schlichting D. (2021). Baricitinib and primary biliary cholangitis. J. Transl. Autoimmun..

[95] Hirschfield G.M., Gershwin M.E., Strauss R., Mayo M.J., Levy C., Zou B., Johanns J., Nnane I.P., Dasgupta B., Li K. (2016). Ustekinumab for patients with primary biliary cholangitis who have an inadequate response to ursodeoxycholic acid: A proof-of-concept study. Hepatology.

[96] Lynch K.D., Chapman R.W., Keshav S., Montano-Loza A.L., Mason A.L., Kremer A.E., Vetter M., de Krijger M., Ponsioen C.Y., Trivedi P. (2019). Effects of vedolizumab in patients with primary sclerosing cholangitis and inflammatory bowel diseases. Clin. Gastroenterol. Hepatol..

[97] De Graaf K.L., Lapeyre G., Guilhot F., Ferlin W., Curbishley S.M., Carbone M., Richardson P., Moreea S., McCune C.A., Ryder S.D. (2018). NI-0801, an anti-chemokine (C-X-C motif) ligand 10 antibody, in patients with primary biliary cholangitis and an incomplete response to ursodeoxycholic acid. Hepatol. Commun..

[98] Tandon P., Rowe B.H., Vandermeer B., Bain V.G. (2007). The Efficacy and Safety of Bile Acid Binding Agents, Opioid Antagonists, or Rifampin in the Treatment of Cholestasis-Associated Pruritus. Am. J. Gastroenterol..

[99] Kuiper E.M., van Erpecum K.J., Beuers U., Hansen B.E., Thio H.B., de Man R.A., Janssen H.L., van Buuren H.R. (2010). The potent bile acid sequestrant colesevelam is not effective in cholestatic pruritus: Results of a double-blind, randomized, placebo-controlled trial. Hepatology.

[100] Shah A., Crawford D., Burger D., Martin N., Walker M., Talley N.J., Tallis C., Jones M., Stuart K., Keely S. (2019). Effects of Antibiotic Therapy in Primary Sclerosing Cholangitis with and without Inflammatory Bowe Disease: A Systematic Review and Meta-Analysis. Semin. Liver Dis..

[101] Elfaki D.A., Lindor K.D. (2011). Antibiotics for the Treatment of Primary Sclerosing Cholangitis. Am. J. Ther..

[102] Damman J.L., Rodriguez E.A., Ali A.H., Buness C.W., Cox K.L., Carey E.J., Lindor K.D. (2018). Review article: The evidence that vancomycin is a therapeutic option for primary sclerosing cholangitis. Aliment. Pharmacol. Ther..

[103] Abarbanel D.N., Seki S.M., Davies Y., Marlen N., Benavides J.A., Cox K., Nadeau K.C., Cox K.L. (2013). Immunomodulatory effect of vancomycin on Treg in pediatric inflammatory bowel disease and primary sclerosing cholangitis. J. Clin. Immunol..

[104] Davies Y.K., Tsay C.J., Caccamo D.V., Cox K.M., Castillo R.O., Cox K.L. (2013). Successful treatment of recurrent primary sclerosing cholangitis after orthotopic liver transplantation with oral vancomycin. Case Rep. Transplant..

[105] Khurana S., Singh P. (2006). Rifampin is safe for treatment of pruritus due to chronic cholestasis: A meta-analysis of prospective randomized-controlled trials. Liver Int..

[106] Hague W.M., Callaway L., Chambers J., Chappel L., Coat S., de Haan-Jebbink J., Dekker M.A., Dixon P., Dodd J., Fuller M. (2021). A multi-centre, open label, randomised, parallel-group, superiority Trial to compare the efficacy of URsodeoxycholic acid with RIFampicin in the management of women with severe early onset Intrahepatic Cholestasis of pregnancy: The TURRIFIC randomised trial. BMC Pregnancy Childbirth.

